# LOTUS: A single- and multitask machine learning algorithm for the prediction of cancer driver genes

**DOI:** 10.1371/journal.pcbi.1007381

**Published:** 2019-09-30

**Authors:** Olivier Collier, Véronique Stoven, Jean-Philippe Vert

**Affiliations:** 1 Modal’X, UPL, Univ Paris Nanterre, F-92000 Nanterre, France; 2 MINES ParisTech, PSL University, CBIO-Centre for Computational Biology, F-75006 Paris, France; 3 Institut Curie, F-75248 Paris Cedex 5, France; 4 INSERM U900, F-75248 Paris Cedex 5, France; 5 Google Research, Brain team, F-75009 Paris, France; National Center for Biotechnology Information (NCBI), UNITED STATES

## Abstract

Cancer driver genes, *i.e.*, oncogenes and tumor suppressor genes, are involved in the acquisition of important functions in tumors, providing a selective growth advantage, allowing uncontrolled proliferation and avoiding apoptosis. It is therefore important to identify these driver genes, both for the fundamental understanding of cancer and to help finding new therapeutic targets or biomarkers. Although the most frequently mutated driver genes have been identified, it is believed that many more remain to be discovered, particularly for driver genes specific to some cancer types. In this paper, we propose a new computational method called LOTUS to predict new driver genes. LOTUS is a machine-learning based approach which allows to integrate various types of data in a versatile manner, including information about gene mutations and protein-protein interactions. In addition, LOTUS can predict cancer driver genes in a pan-cancer setting as well as for specific cancer types, using a multitask learning strategy to share information across cancer types. We empirically show that LOTUS outperforms five other state-of-the-art driver gene prediction methods, both in terms of intrinsic consistency and prediction accuracy, and provide predictions of new cancer genes across many cancer types.

## Author summary

Cancer development is driven by mutations and dysfunction of important, so-called cancer driver genes, that could be targeted by specific therapies. While a number of such cancer genes have already been identified, it is believed that many more remain to be discovered. To help prioritize experimental investigations of candidate genes, several computational methods have been proposed to rank promising candidates based on their mutations in large cohorts of cancer cases, or on their interactions with known driver genes in biological networks. We propose LOTUS, a new computational approach to identify genes with high oncogenic potential. LOTUS implements a machine learning approach to learn an oncogenic potential score from known driver genes, and brings two novelties compared to existing methods. First, it allows to easily combine heterogeneous sources of information into the scoring function, which we illustrate by learning a scoring function from both known mutations in large cancer cohorts and interactions in biological networks. Second, using a multitask learning strategy, it can predict different driver genes for different cancer types, while sharing information between them to improve the prediction for every type. We provide experimental results showing that LOTUS significantly outperforms several state-of-the-art cancer gene prediction software.

## Introduction

In our current understanding of cancer, tumors appear when some cells acquire functionalities that give them a selective growth advantage, allowing uncontrolled proliferation and avoiding apoptosis [1, 2]. These malignant characteristics arise from various genomic alterations including point mutations, gene copy number variants (CNVs), translocations, inversions, deletions, or aberrant gene fusions. Many studies have shown that these alterations are not uniformly distributed across the genome [3, 4], and target specific genes associated with a limited number of important cellular functions such as genome maintenance, cell survival, and cell fate [5]. Among these so-called *driver genes*, two classes have been distinguished in the literature: *tumor suppressors genes* (TSGs) and *oncogenes* (OGs) [6, Chapter 15]. TSGs, such as TP53 [7], participate in defense mechanisms against cancer and their inactivation by a genomic alteration can increase the selective growth advantage of the cell. On the contrary, alterations affecting OGs, such as KRAS [8] or ERBB2 [9], can be responsible for the acquisition of new properties that provide some selective growth advantage or the ability to spread to remote organs. Identifying driver genes is important not only from a basic biology point of view to decipher cancer mechanisms, but also to identify new therapeutic strategies and develop precision medicine approaches targeting specifically mutated driver genes. For example, Trastuzumab [10] is a drug given against breast cancer that targets the protein precisely encoded by ERBB2, which has dramatically improved the prognosis of patients whose tumors overexpress that OG.

Decades of research in cancer genomics have allowed to identify several hundreds of such cancer genes. Regularly updated databases such as the Cancer Gene Census (CGC) [11], provide catalogues of genes likely to be causally implicated in cancer, with various levels of experimental validations. Many cancer genes have been identified recently by systematic analysis of somatic mutations in cancer genomes, as provided by large-scale collaborative efforts to sequence tumors such as The Cancer Genome Atlas (TCGA) [12] or the International Cancer Genome Consortium (ICGC) [13]. Indeed, cancer genes tend to be more mutated than non-cancer genes, providing a simple guiding principle to identify them. In particular, the COSMIC database [14] is the world’s largest and most comprehensive resource of somatic mutations in coding regions. It is now likely that the most frequently mutated genes have been identified [15]. However, the total number of driver genes is still a debate, and many driver genes less frequently mutated, with low penetrance, or specific to a given type of cancer are still to be discovered.

The first methods to identify driver genes from catalogues of somatic mutations simply compared genes based on somatic mutation frequencies, which was proved to be far too basic [16]. Indeed, mutations do not appear uniformly on the genome: some regions of the genome may be more affected by errors because they are more often transcribed, so that some studies actually overestimated the number of driver genes because they were expecting lower mutation rates than in reality. Mathematically, they were formulating driver prediction as a hypothesis testing problem with an inadequate null hypothesis [17]. Several attempts have been made to adequately calibrate the null hypothesis, like [16] or [18], where it is assumed that mutations result from a mixture of several mutational processes related to different causes.

A variety of bioinformatics methods have then been developed to complete the list of pan-cancer or cancer specific driver genes. Globally, they fall into three main categories. First, a variety of “Mutation Frequency” methods such as MuSiC [19] or ActiveDriver [20] identify driver genes based on the assumption that they display mutation frequencies higher than those of a background mutation model expected for passenger mutations. However, this background rate may differ between cell types, genome positions or patients. In order to avoid such potential bias, some methods like MutSigCV [21] derive a patient-specific background mutation model, and may take into account various criteria such as cancer type, position in the genome, or clinical data. Second, “Functional impact” methods such as OncodriveFM [22] assume that driver genes have higher frequency of mutations expected to impact the protein function (usually missense mutations) than that observed in passenger genes. Third, “Pathway-based” methods consider cancer as a disease in which mutated genes occupy key roles in cancer-related biological pathways, leading to critical functional perturbations at the level of networks. For example, DriverNet [23] identifies driver genes based on their effect in the transcription networks. Although these methods tend to successfully identify the most frequently mutated genes, their overall prediction overlap is modest. Since they rely on complementary statistical strategies, one could recommend to use them in combination, as CompositeDriver allows us to do [24]. The results of some of these tools are available at the Driver DB database [25].

Some methods integrate information on mutation frequency and functional impact of mutations, or other types of data such as genome position, copy number variations (CNVs) or gene expression. The underlying idea is that combining data should improve the prediction performance over tools that use a single type of information. For example, TUSON [26] or DOTS-Finder [27] combine mutation frequencies and functional impact of mutations to identify OGs and TSGs. Also in this category, the 20/20+ method [28] encodes genes with features based on their frequency and mutation types, in addition to other biological information such as gene expression level in difference cancer cell lines [29] or replication time. Then, 20/20+ predicts driver genes with a random forest algorithm, which constitutes the first attempt to use a machine learning method in this field. In [28], the authors benchmark 8 driver gene prediction methods based on several criteria including the fraction of predicted genes in CGC, the number of predicted driver genes and the consistency. Three methods proved to perform similarly on all criteria, and better than the five others: TUSON, MutSigCV, and 20/20+, validating the relevance of combining heterogeneous information to predict cancer genes.

In the present paper, we propose a new method for cancer driver gene prediction called *Learning Oncogenes and TUmor Suppressors* (LOTUS). Like 20/20+, LOTUS is a machine learning-based method, meaning that it starts from a list of known driver genes in order to “learn” the specificities of such genes and to identify new ones. In addition, LOTUS presents two unique characteristics with respect to previous work in this field. First, it combines three types of features likely to contain relevant information to predict cancer genes (mutation frequency, functional impact, and pathway-based informations). This integration of heterogeneous information is carried out in a unified mathematical and computational framework thanks to the use of kernel methods [30], and allows in principle to integrate other sources of data if available, such as transcriptomic or epigenomic information. More precisely, in our implementation, we predict cancer driver genes based not only on gene mutations features like “Mutation Frequency” and “Functional Impact” methods do, but also on known protein-protein interaction (PPI) network like “Pathway-based” methods do. Indeed, the use of PPI information is particularly relevant since it has been reported that proteins encoded by driver genes are more likely to be involved in protein complexes and share higher “betweenness” than a typical protein [26]. Moreover, it has been successfully used by HotNet2 [31] to detect gene pathways enriched in driver genes, and in [32] for cancer driver prediction. Second, LOTUS can predict cancer genes in a pan-cancer setting, as well as for specific cancer types, using a multitask learning strategy [33].

Although many efforts are devoted to identify cancer-specific genes based on experimental approaches, in in-silico approaches, the pan-cancer setting has been adopted by most available prediction methods, since more data are available to train models when gathering all cancer types. Prediction of drivers for specific cancer types has been less explored so far, because the number of known driver genes for a given cancer is often too small to build a reliable prediction model, and because the amount of data such as somatic mutations to train the model is smaller than in the pan-cancer setting. However, the search for cancer specific driver genes is relevant, because cancer is a very heterogeneous disease: different tumorigenic processes seem to be at work in different tissue types, and consequently, each cancer type probably has its own list of driver genes [15]. LOTUS implements a multitask algorithm that predicts new driver genes for a given cancer type based on its known driver genes, while also taking into account the driver genes known for other types of cancer according to their similarities with the considered type of cancer. Such approaches are of particular interest when the learning data are scarce in each individual tasks: they increase the amount of data available for each task and thus perform statistically better. To our knowledge, while a similar approach was used to predict disease genes across general human diseases [34], this is the first time a multitask machine learning algorithm is used for the prediction of cancer driver genes.

We compare LOTUS to five state-of-the art cancer prediction methods. We show that LOTUS outperforms the state-of-the-art in its ability to identify novel cancer genes, and clarify the benefits of heterogeneous data integration and of the multitask learning strategy to predict cancer type-specific driver genes. Finally, we provide predictions of new cancer genes according to LOTUS, as well as supporting evidence that those predictions are likely to contain new cancer genes.

## Results

### LOTUS, a new method for pan-cancer and cancer specific driver gene prediction

We propose LOTUS, a new method that predicts cancer driver genes. LOTUS is a machine learning-based method that estimates a scoring function to rank candidate genes by decreasing probability for them to be OGs or TSGs, given a training set of known OGs and TSGs. The score of a candidate gene is a weighted sum of similarities between the candidate gene and the known driver genes, where the weights are optimized by a one-class support vector machine (OC-SVM) algorithm. The similarities between genes are calculated based on gene features that are derived from the analysis of somatic mutation patterns in the genes (see Materials ans methods section for a description of these features), or from the relative positions of genes in a PPI network, or from both; the mathematical framework of kernel methods allows to simply combine heterogeneous data about genes (i.e., patterns of somatic mutations and PPI information) in a single model.

Another salient feature of LOTUS is its ability to work in a pan-cancer setting, as well as to predict driver genes specific to individual cancer types. In the later case, we use a multitask learning strategy to jointly learn scoring functions for all cancer types by sharing information about known driver genes in different cancer types. We test both a default multitask learning strategy, that shares information uniformly across all cancer types, and a new strategy that shares information across cancer types according to their degree of similarity. More details about the mathematical formulation and algorithms implemented in LOTUS are provided in the Material and Methods section.

In the following, we assess the performance of LOTUS first in the pan-cancer regime, i.e. in the single task setting, and compare it to five state-of-the-art methods (TUSON, MutSigCV, 20/20+, MUFFINN and DiffMut), and second in the cancer type specific regime, where we illustrate the importance of the multitask learning strategies.

### Cross-validation performance for pan-cancer driver gene prediction

We first study the pan-cancer regime, where cancer is considered as a single disease, and where we search for driver genes involved in at least one type of cancer. Several computational methods have been proposed to solve this problem in the past, and we compare LOTUS with five well-known state-of-the-art methods [28]: MutSigCV [21], which is a frequency-based method, TUSON [26], 20/20+ [28], which combines frequency and functional information, MUFFINN [32] and DiffMut [35], which takes the mutation patterns of genes into account.

As explained in the *Materials and methods* section, to perform a fair comparison between different methods we use different training databases for LOTUS adapted to each competing methods. We measure the performance of each method in terms of consistency error (*CE*), which estimates the mean number of non-driver genes that a given prediction method ranks higher than known driver genes; hence the smaller the *CE* the better the method. The results in terms of cross-validated *CE* for OG and TSG prediction are presented in Table 1 for TUSON and MUFFINN, in Table 2 for 20/20+ and DiffMut, and in Table 3 for MutSigCV. When analyzing these results, one should keep in mind that the total number of cancer driver genes is still a subject of debate, but it is expected to be much lower than the size of the test set (which depends on the method but is of the order of 18, 000), and it should rather be in the range of a few hundreds. Therefore, *CE* above a few thousand can be considered as poor performance results.

**Table 1:** Comparison of TUSON, MUFFINN and LOTUS in the pan-cancer cross-validation regime. This table shows the mean cross-validated *CE* for OG and TSG prediction by TUSON, MUFFINN and LOTUS, trained on the TUSON database.

Driver type ∖ Method	TUSON	MUFFINN	LOTUS
OG	3,286	1,924	**990**
TSG	626	678	**127**

**Table 2:** Comparison of 20/20+, DiffMut and LOTUS in the pan-cancer cross-validation regime. This table shows the mean cross-validated *CE* for OG and TSG prediction by 20/20+, DiffMut and LOTUS, trained on the 20/20+ database.

Driver type ∖ Method	20/20+	DiffMut	LOTUS
OG	1,831	4,254	**782**
TSG	845	2,537	**468**

**Table 3:** Comparison of MutSigCV and LOTUS in the pan-cancer cross-validation regime. This table shows the mean cross-validated *CE* for OG and TSG prediction by MutSigCV and LOTUS, trained on the MutSigCV database.

Driver type ∖ Method	MutSigCV	LOTUS
OG	6,294	**1,929**
TSG	7,232	**2,990**

These results show that LOTUS strongly outperforms all other algorithms in term of *CE*, for both TSG and OG predictions. More precisely, for OG predictions, LOTUS is about three times better than MutSigCV and TUSON, twice better than 20/20+ and MUFFINN, and five times better than DiffMut, in terms of *CE*. For TSG predictions, the reduction in *CE* with LOTUS is two-fold compared to MutSigCV and 20/20+, and five-fold compared to TUSON, MUFFINN DiffMut. Note that the performance is overall much better in the first two experiments, which are also easier because they provide larger mutational data.

It is interesting to note that, for all methods except in the MutSigCV experiment, the performances obtained for OG do not reach those obtained for TSG, suggesting that OG prediction is a more difficult problem than TSG prediction. This reflects the fundamental difference between TSG mutations and OG mutations: the first lead to loss-of-function and can pile up, while the second are gain-of-function mutations and have a much more subtle nature. In addition, gain-of-function can also be due to overexpression of the OG, which can arise from other mechanisms than gene mutation. One way to improve the OG prediction performance may be to include descriptors better suited to them, such as copy number. Moreover, as mutations affecting OGs are not all likely to provide them with new functionalities, many mutations on OGs present in the database and used here might not bear information on OGs. Therefore, relevant information on OGs is scarce, which makes OG prediction more difficult. In addition, the data themselves might also contribute to difference in performance between TSG and OG prediction. For example, in the case of the TUSON train set, although the TSG and OG train sets both contain 50 genes, the mutation matrix that we used to build the gene features contains 13, 525 mutations affecting TSGs and 7, 717 mutations affecting OGs. Therefore, the data are richer for TSG, which might contribute to the difference in prediction performance.

### The benefits of combining mutations and PPI informations

LOTUS, 20/20+, MutSigCV, DiffMut, MUFFINN and TUSON differ not only by the algorithm they implement, but also by the type of data they use to make predictions: in particular, TUSON and 20/20+ use only mutational data while LOTUS uses PPI information in addition to mutational data. To highlight the contributions of the algorithm and of the PPI information to the performance of LOTUS, we ran LOTUS with *K*_*genes*_ equal to *K*_*mutation*_ or *K*_*PPI*_, *i.e.*, with only mutation information, or only PPI information.

The results are presented in the first two columns of Tables 4 and 5, respectively for OG and TSG. The last column of these tables recalls the performance obtained when mutation and PPI information are both used (values reported from Tables 1, 2 and 3).

**Table 4:** Performance of LOTUS for OG prediction with different gene kernels.

Train set ∖ Kernel	*K*_*mutation*_	*K*_*PPI*_	*K*_*degree*_	*K*_*mutation*_ + *K*_*degree*_	*K*_*mutation*_ + *K*_*PPI*_
TUSON datasets	2,904	1,574	1,659	1,553	**990**
20/20 datasets	2,453	1,642	1,774	1,628	**782**
MutSig datasets	2,292	1,450	1,306	**1,274**	1,929

This table shows the *CE* of LOTUS for OG prediction in the pan-cancer setting, with different gene kernels (columns) and different gold standard sets of known OGs and mutations (rows).

**Table 5:** Performance of LOTUS for TSG prediction with different gene kernels.

Train set ∖ Kernel	*K*_*mutation*_	*K*_*PPI*_	*K*_*degree*_	*K*_*mutation*_ + *K*_*degree*_	*K*_*mutation*_ + *K*_*PPI*_
TUSON datasets	393	1,413	1,965	1,669	**127**
20/20 datasets	971	2,460	2,994	2,392	**468**
MutSig datasets	4,335	4,017	4,253	3,818	**2,990**

This table shows the *CE* of LOTUS for TSG prediction in the pan-cancer setting, with different gene kernels (columns) and different gold standard sets of known OGs and mutations (rows).

These results show that, both for OG and TSG, using both mutation and PPI information dramatically improves the prediction performance over using only one of them. This underlines the fact that mutation and PPI bear complementary information that are both useful for the prediction tasks. The performances obtained with only PPI information are similar for OG and TSG, which seems to indicate that this information contributes similarly to both prediction tasks. On the contrary, the performances obtained using only mutation information are much better for TSG than for OG. This is consistent with the above comment that mutation information is more abundant in the database and more relevant in nature for TSG than for OG. It is also consistent with the fact that using *K*_*mutation*_ alone outperforms using *K*_*PPI*_ alone for TSGs, while the opposite is observed for OGs.

**Fig 1:**
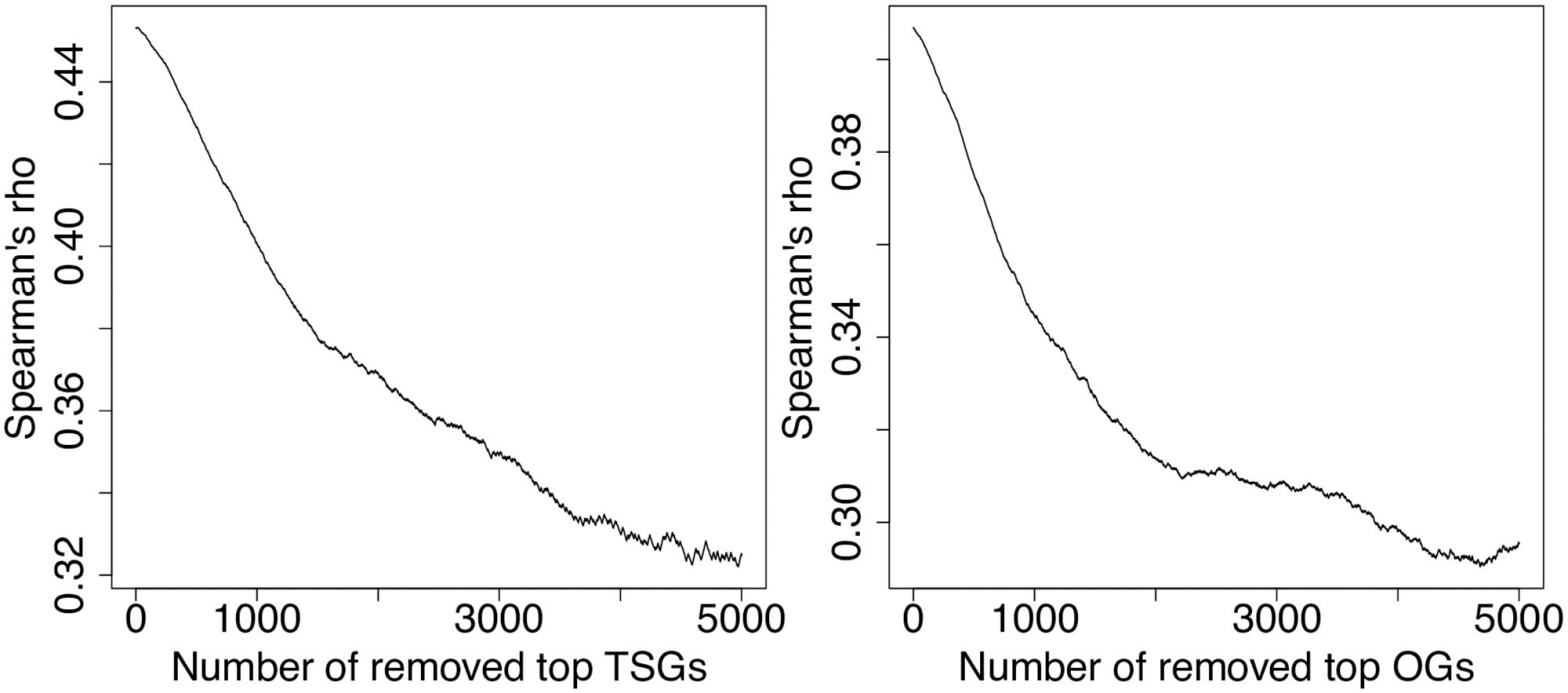
Correlation between gene degree and LOTUS rank. This plot shows the Spearman’s rank correlation between gene degrees in the PPI network and LOTUS ranks (when trained with the 20/20 dataset), as a function of how many top-ranked TSGs (left) and OGs (right) are removed before computing the correlation.

A possible issue with PPI network-based models is that they may be subject to study bias, in the sense that cancer genes tend to be more studied and thus have higher degree in the PPI network. Hence learning an association between cancer genes and degree in a PPI network may be problematic since this is not purely a biological feature, and we may not be able to extrapolate the model to new, less studied cancer genes. This concern is supported by the observation that the gene rank computed by LOTUS with PPI kernel significantly correlates with the gene degree in the PPI network (Fig 1). This correlation only slowly decreases when one removes the top genes in the list, which indicates that this phenomenon can not only be attributed to a few genes (as opposed to, e.g., supplementary Figure 22 in [31]).

To assess whether the good performance of LOTUS using PPI is only due to the gene degree information, we performed two further experiments. First, we ran LOTUS with the kernel *K*_*degree*_ defined by:

$$\begin{array}{c} {\left(K_{degree}\right)}_{i,j}=d_{i}d_{j}, \end{array}$$

where *d*_*i*_ is the degree of *i* in the PPI network. This allows LOTUS to capture in its model a linear function of the degree. We see in Tables 4 and 5 that the degree kernel has in almost all cases worse performance than the PPI kernel, which confirms that the number of neighbors alone contains less relevant information in relation with the driver prediction problem than the PPI kernel does. Second, we trained LOTUS with a PPI kernel derived from a randomized PPI network, where we kept the network structure but randomly shuffled the genes while approximately preserving their degree. For that purpose, we binned the genes in 20 groups of roughly equal sizes, by decreasing degree in the network, and randomly shuffled the genes within each group. After this random shuffling, each gene has approximately the same degree as in the initial PPI network, but not the same neighborhood. We repeated the random shuffling 100 times and computed the performance of LOTUS with the corresponding PPI kernels. We observed that the performance was worse with the randomized PPI network than with the original PPI network in 95% of the cases, confirming that LOTUS with the PPI kernel uses more than the mere degree information to predict cancer genes.

Furthermore, we examined the first predictions (excluding already known driver genes) of LOTUS with the 20/20 datasets, when both the mutation and the PPI kernels are used. Among the first 50 TSGs (described by the number of frameshift, LOF and splice mutations), 26 have less than 3 mutations of each kind, 4 predicted TSGs even having no mutation at all. This demonstrates that LOTUS predictions strongly benefit from the PPI information, and that some of these genes would have never been detected using mutation data only.

Finally, note that gene lengths and PPI-network degrees are not used explicitly as features by LOTUS, although these characteristics correlate with our predictions. Hence, LOTUS retrieves implicit relevant characteristics of cancer driver genes from the mutations and the PPI-network.

### Performance on CGCv86 prediction in the pan-cancer regime

**Fig 2:**
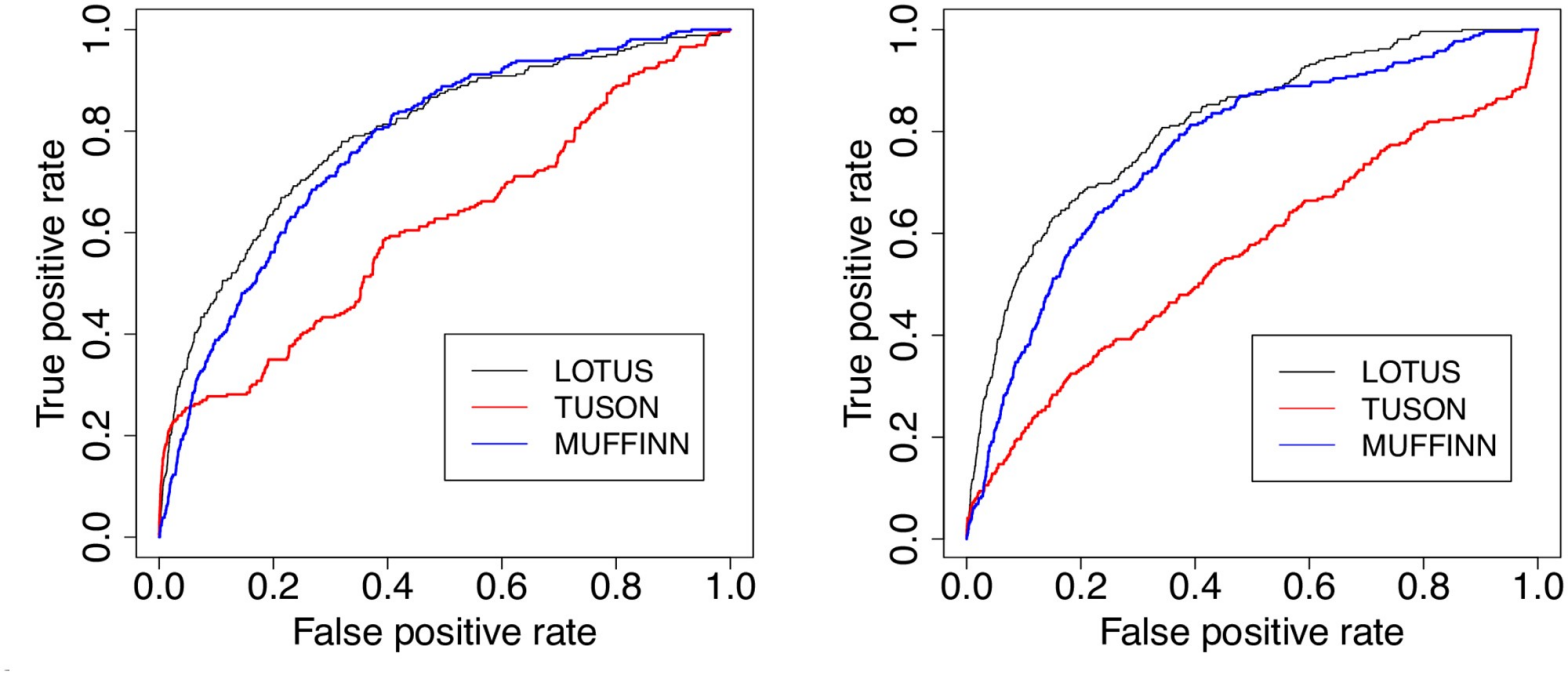
ROC curves of different cancer gene prediction methods trained on the TUSON database. ROC curves of different methods for TSG (left) and OG (right) prediction when trained on the TUSON database, and evaluated on the discovery of cancer genes recently added in CGCv86.

**Fig 3:**
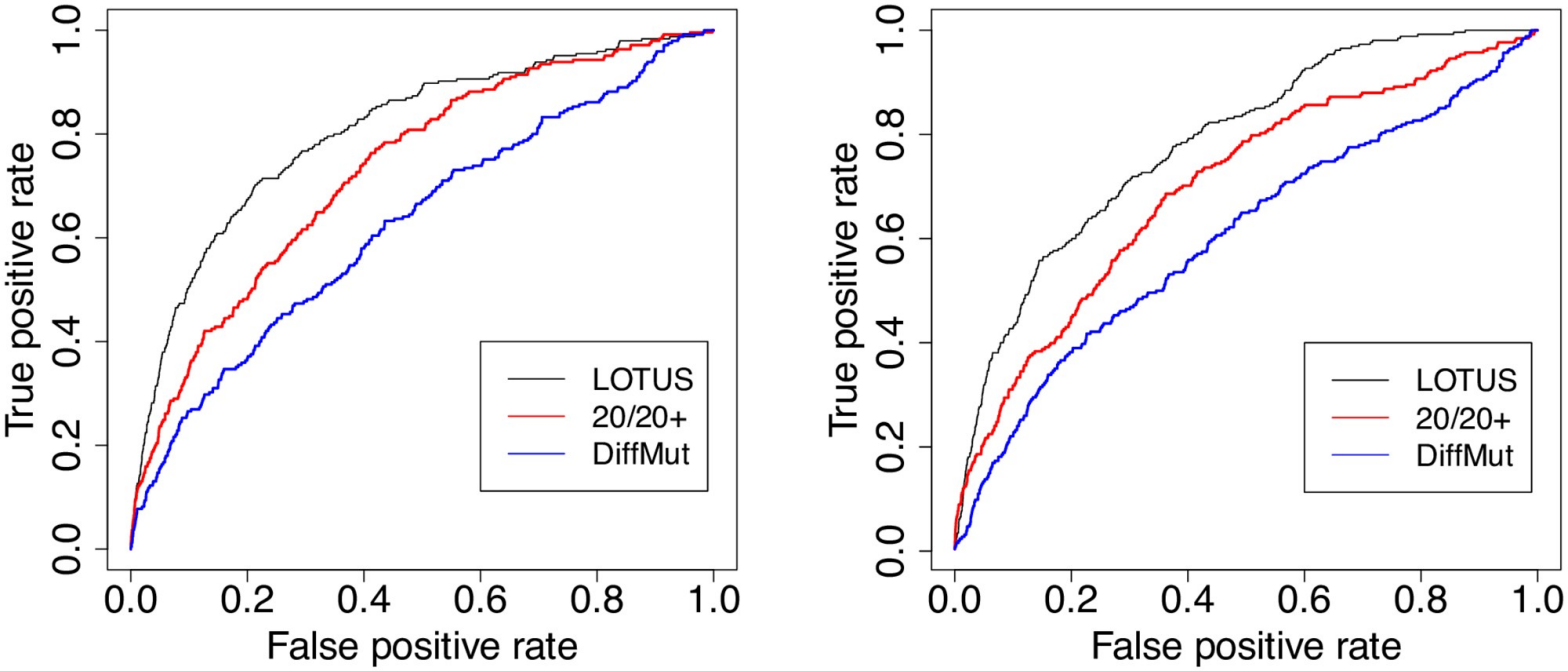
ROC curves of different cancer gene prediction methods trained on the 20/20 database. ROC curves of different methods for TSG (left) and OG (right) prediction when trained on the 20/20 database, and evaluated on the discovery of cancer genes recently added in CGCv86.

We now evaluate the generalization properties of the different methods on new unseen data as external test set. This could mitigate the potential bias in the evaluation of the performance of TUSON, DiffMut and 20/20+ based on cross-validation experiments, as in the previous paragraph. For that purpose, we evaluate the performance of the different methods when predicting supposedly “difficult” new cancer genes (an independent test set), which have only been added recently in CGCv86. We train on the one hand LOTUS, MUFFINN and TUSON with the TUSON mutation database and driver gene train sets, and on the other hand LOTUS, DiffMut and 20/20+ with the 20/20 mutation database and driver gene train sets. Then, we make predictions on the remaining genes in COSMIC, and count how many driver genes in CGCv86 appear among the 20, 50 and 100 first predictions. Note that the driver genes from the train sets were excluded from the predictions. The results are shown in Tables 6–9 and are illustrated by corresponding receiver operating characteristic (ROC) curves in Figs 2 and 3.

**Table 6:** Performance of different methods trained on the TUSON database for TSG prediction in CGCv86.

Method ∖ Number of predictions	20	50	100
MUFFINN	3	6	13
LOTUS	5	10	19
TUSON	**13**	**25**	**35**

For each method (row) trained on the TUSON database, the table shows the number of recently added TSG in CGCv86 predicted among the top *k* predictions, for *k* = 20, 50 or 100 (columns).

**Table 7:** Performance of different methods trained on the TUSON database for OG prediction in CGCv86.

Method ∖ Number of predictions	20	50	100
MUFFINN	3	5	11
LOTUS	3	7	**16**
TUSON	**7**	**11**	12

For each method (row) trained on the TUSON database, the table shows the number of recently added OG in CGCv86 predicted among the top *k* predictions, for *k* = 20, 50 or 100 (columns).

**Table 8:** Performance of different methods trained on the 20/20+ database for TSG prediction in CGCv86.

Method ∖ Number of predictions	20	50	100
LOTUS	4	**10**	**16**
DiffMut	1	4	10
20/20+	**7**	9	**16**

For each method (row) trained on the 20/20+ database, the table shows the number of recently added TSG in CGCv86 predicted among the top *k* predictions, for *k* = 20, 50 or 100 (columns).

**Table 9:** Performance of different methods trained on the 20/20+ database for OG prediction in CGCv86.

Method ∖ Number of predictions	20	50	100
LOTUS	3	5	9
DiffMut	2	3	4
20/20+	**10**	**15**	**20**

For each method (row) trained on the 20/20+ database, the table shows the number of recently added OG in CGCv86 predicted among the top *k* predictions, for *k* = 20, 50 or 100 (columns).

First, we observe that TUSON outperforms LOTUS in almost all these experiments. Second, LOTUS outperforms DiffMut and MUFFINN in all experiments. Third, LOTUS is better than 20/20+ for TSG detection, and the contrary holds for OGs. Generally speaking, the first predictions of TUSON and 20/20+ are more reliable than LOTUS’s, but, as shown in Figs 2 and 3, LOTUS outperforms all the methods when all genes are considered, and not only the first 20 to 100 genes.

The good performance of TUSON and 20/20+ for the top ranked genes compared to those of LOTUS could be explained by the fact that, all genes in CGCv86 so far have been reported through analysis of mutation data (cf. CGC web page: ‘The Cancer Gene Census (CGC) is an ongoing effort to catalogue those genes which contain mutations that have been causally implicated in cancer’). This interpretation would also explain why LOTUS hardly agrees with the other methods when comparing the top ranked genes. Indeed, for the 20 top predictions of LOTUS (excluding training sets), the intersection with TUSON consists only in two TSGs and one OG, the intersection with DiffMut consist of one TSG and two OGs, and the intersection with 20/20+ consists in four TSGs and one OG. Since LOTUS and MUFFINN are the only methods, among those considered here, that use network information in addition to mutation data, this could explain less overlap between their predictions and those of the other methods, and a lower overlap with CGC for the top ranked genes, since no PPI information was used to establish the CGC database.

To underpin this hypothesis, we computed the total number of non-silent mutations for the 20, 50 and 100 first predictions for all method, *i.e.*, the top-ranked genes not belonging to the training sets. The result in Tables 10 and 11 show that predictions from LOTUS have lower mutations rates than those of TUSON, 20/20+ and DiffMut, especially for TSGs. This demonstrates that LOTUS relies less on mutation data than these methods. Similarly, MUFFINN, that also uses network information, tends to rank genes with fewer mutations on top of the list.

**Table 10:** Total number of non-silent mutations in the top predicted genes by different methods trained on the TUSON database.

Driver type ∖ Method	TUSON	LOTUS	MUFFINN
20 first OG	**2**	3	4
50 first OG	3	3	**2**
100 first OG	**3**	**3**	7
20 first TSG	30	7	**4**
50 first TSG	14	5	**2**
100 first TSG	11	5	**4**

This table shows the total number of non-silent mutations in the top-ranked genes predicted by different method trained on the TUSON database (columns), for both OG and TSG (rows).

**Table 11:** Total number of non-silent mutations in the top predicted genes by different methods trained on the 20/20+ database.

Driver type ∖ Method	DiffMut	20/20+	LOTUS
20 first OG	25	**1**	**1**
50 first OG	17	**1**	**1**
100 first OG	11	**1**	**1**
20 first TSG	25	11	**2**
50 first TSG	18	9	**3**
100 first TSG	14	6	**3**

This table shows the total number of non-silent mutations in the top-ranked genes predicted by different method trained on the TUSON database (columns), for both OG and TSG (rows).

### Analysis of new driver genes predicted by LOTUS

We tested the ability of LOTUS to make new driver gene predictions. We trained LOTUS with the CGCv86 train set, made predictions over the complete COSMIC database (19,320 genes including the training sets). The complete results are given in S1 Table. Complete analysis of the predicted OGs and TSGs rankings is out of the scope of this paper. However, we considered the 22/21 best ranked TSGs and OGs, and made bibliographic search in order to look for independent information that could validate these predictions.

For the 22 best ranked predicted OGs, we found abundant literature reporting their implication in various cancers. It is not possible to make a full review for each of these genes, but we group them based on their global functions and focus on some examples.

Twelve out of the 22 best ranked genes are related to transcription regulation, a mechanism that is invariably perturbed in cancer. These 12 genes are involved at various levels of transcription regulation: chromatin remodeling (PYGO1, PYGO2, EP300, DOT1L), transcription factors or repressors (MSEI1, MSEI2, MSEI3, TFEC, NKX2-2, ZIK1), transcription regulation via miRNA (DROSHA, ELF1). For all these genes, a corpus of publications confirm their role in promoting diverse types of cancers. For example, PYGO1 is involved in colorectal cancer [36], while PYGO2 was shown to be a tumor promoter in mice [37]. MSEI1, MSEI2, and MSEI3 are involved in the etiology, progression and metastatic evolution of some cancer types such as prostate cancer [38], or leukemia [39, 40]. DROSHA is involved in the miRNA depletion observed in lung cancer, and alterations in this gene was shown to have a remarkable impact in lung cancer [36].

Eight other best ranked OGs belong to signaling pathways known to play a role in cancer. Among them, FGF6 and FGF5 are growth factors from the fibroblast growth factors signaling pathway, which are well known players contributing to tumor progression [41]. Similarly MOB3B (or MOB1) is a pivotal kinase player in the Hippo tumor suppressor pathway, and mutations in this gene is associated to prostate cancer susceptibility and agressive tumors [42].

Although not exhaustive, these findings indicate that the best ranked oncogenes predicted by LOTUS are realistic OG for some cancer types.

As for OGs, for the 21 best ranked TSGs, we found many publications indicating their role in cancer, and strikingly, APOM is actually a known TSG for hepatocellular carcinoma [43]. We will group some of the other TSGs based on their function, and discuss a few examples. Five genes are involved in DNA repair, a role closely related to genome maintenance and cancer [44, 45], and were shown to have a protective role in various types of cancer (only one paper is cited by gene, but many others confirm this role): EXO1 [46], ERCC1 [47], GTF2H1 [48], MDC1 [49], and DGCR8 [50].

Three genes are involved in immune system response to cancer, which clearly indicates that they bear a protective role: PDCD1 [50], KLRG1 [51], and MUC16 [52].

For six other genes of various functions, we found recent publications indicating that they could potentially act as TSGs: SPTA1, GALNT5, PIWIL1, PIWIL4, SNX5, ADAM6. Mutations in SPTA1 was found to play a role in glioblastoma [53]. The expression of the 5 other genes were found to be repressed by over-expressed non-coding RNA or by aberrant methylation of the promoter: GALNT5 in gastric cancer [54], ADAM6 in lung adenocarcinoma [55], PIWIL1 and PIWIL4 in lung adenocarcinoma [56] and renal cell carcinoma [57]. Loss or decreased expression SNX5 promotes thyroid cancer progression [58].

Intriguingly, 3 of the best 21 ranked predicted TSGs, bibliographic search provided clues that they indeed play a role in cancer, but that they would rather behave as OG. These genes are CENPU [59], FXYD2 [60], ANXA9 [61]. In fact, the literature provides other examples of genes able to switch from oncogenes to tumor suppressor genes, depending on the context [62], which could be the case for these genes. In most cases, the cited papers (and others) observe that over-expression of these genes are observed in various types of cancers. One assumption could be that variations of their levels of expression might lead to switch between TSG and OG roles.

Interestingly, among the 50 best ranked TSGs, 4 genes are not mutated (CENPU, AEBP2, CDX1, ZNF652), and 22 genes are rarely mutated, less than 3 times. Such cases are not observed among the top ranked OGs. Indeed, in the case of TSG, decrease in expression or gene deletion leads to the same effect, i.e. loss of function, as deleterious mutations within the gene sequence. A TSG that loses its function based on default in expression cannot be retrieved by prediction methods based only on mutations, and LOTUS probably classified these rarely mutated genes probably according to their interactions in the PPI network. The case of CENPU is already discussed above. We could confirm that CDX1 is a known TSG [63]. Silencing of AEBP2 with long non coding RNA is associated to melanoma [64], while its deletion is observed in myeloid leukemia [65]. Similarly, silencing of ZNF652 by miRNA is involved in lymphoma [66], while some genetic variants of this gene lead to higher risks of prostate cancer [67]. Concerning the 22 rarely mutated TSGs, 9 of them are already discussed above, and most of the others are known to play a role in cancer. As an example, TNIP2 may be a TSG in pancreatic tumors, since it was shown that inhibition of MiR-1180, a short non coding mi-RNA over-expressed in pancreatic cancer targeting TNIP2, inhibited cell proliferation [68].

Taken together, these results show that, among the top TSG and OG ranked by LOTUS, many genes are indeed involved in cancer, and that LOTUS predictions correspond to relevant genes that are reliable candidates as cancer driver genes, at least for some tumor types.

### Identification of cancer-specific driver genes with multitask LOTUS

In this section, we do not consider cancer as a single disease, but as a variety of diseases with different histological types that can affect various organs. Indeed, an important question in cancer research is to identify driver genes for each type of cancer. One way to solve this problem is to use a prediction method that is trained only with driver genes known for the considered cancer. Such single-task methods may however display poor performance because the number of known drivers per cancer is often too small to derive a reliable model. Indeed, scarce training data lead to a potential loss of statistical power as compared to the problem of identification of pan-cancer driver genes where data available for all cancers are used.

In this context, we investigate two multitask versions of LOTUS, where we predict driver genes for a given cancer based on the drivers known for this cancer but also on all driver genes known for other cancer types. For a given cancer type, this may improve driver genes prediction by limiting the loss of statistical power compared to the aforementioned single-task approach.

**Fig 4:**
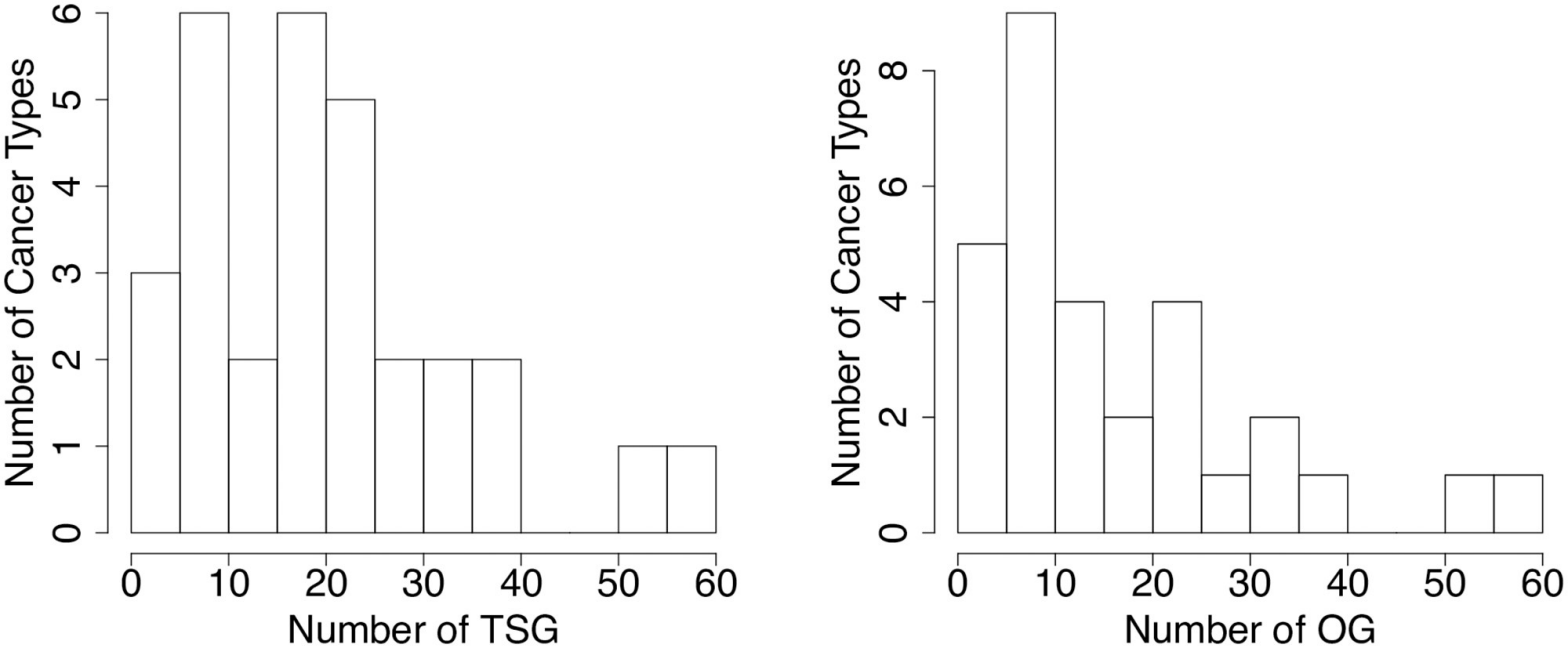
Number of cancer genes per cancer type. This plot shows the distribution of the number of TSGs (left) and OGs (right) per cancer type. For example, 3 cancer types have 0 to 5 TSG, 6 other cancer types have 6 to 10 TSG, etc.

For that purpose, we derive a list of 30 cancer diseases from the 20/20 mutation dataset as explained in Methods. This complete list is available in S2 Table. As expected, many cancer types have only few known cancer genes (Fig 4).

Since we want to evaluate the performance of LOTUS in a cross-validation scheme, we only consider diseases with more than 4 known driver genes in order to be able to run a 2-fold cross-validation scheme. This leads us to keep 27 cancer types for TSG prediction and 27 for OG prediction. Note however that, for each cancer type, prediction are made while sharing all the driver genes known for the 30 diseases, according to their similarities with these cancer types.

The 2-fold cross-validated *CE* of LOTUS for the 27 considered cancer types is presented in Table 12 (for TSG) and Table 13 (for OG). We compare four variants of LOTUS, as explained in Methods: (1) single-task LOTUS treats each disease in turn independently from the others using only the mutation data related to the considered disease to calculate gene features, and only the driver genes known for this disease are used to train the algorithm; (2) Aggregation LOTUS is also a single-task version of LOTUS, but gene features are calculated using the complete mutation database of gene mutations in all cancers. In addition, for each disease, the train set consists of known drivers for all the other cancers and have of the drivers known for the considered disease. Then the prediction performance are calculated for the other half of known drivers for this disease, which constitute the test set in the 2-fold cross validation scheme. Therefore, Aggregation LOTUS is a single-task algorithm that uses much richer information than the basic Single-task LOTUS; (3 and 4) Two multitask versions of LOTUS use either a standard multitask strategy that does not take into account the relative similarities between diseases (Multitask LOTUS), or a more refined multitask strategy where driver gene information is shared between cancer types according to their similarity based on biological information (Multitask LOTUS2). Finally, we compare these performances with those of DiffMut, as a single-task method using only the mutation data related to the considered disease, as for single-task LOTUS.

**Table 12:** Cross-validated *CE* for DiffMut and LOTUS prediction of disease specific TSGs in the single- and multitask settings.

Disease	Number of TSGs	DiffMut	Single-Task LOTUS	Aggregation LOTUS	Multitask LOTUS	Multitask LOTUS2
ALL	38	7,122	1,431	783	709	**631**
Astrocytoma	17	7,605	2,612	49	38	**0**
BladUroCarc	9	4,852	2052	173	138	**115**
BreastAdeno	34	3,250	1,837	801	**770**	778
CLL	21	4,253	1,336	895	**894**	921
Colorectal	53	7,600	3,640	911	870	**825**
EndomCarc	18	5,831	1,222	89	82	**59**
GliobMulti	24	4,776	2,771	166	191	**153**
HNSC	24	5,819	3,051	681	**571**	595
Kidney Cancer	19	6,133	2,766	2,512	**2,474**	**2,474**
LAML	56	5,947	1,936	1,483	1,451	**1,328**
LiverHepCarc	10	3,768	602	221	**156**	172
Low-Grade Glioma	17	6,047	2,712	49	38	**0**
LungAdeno	30	6,773	4,712	339	341	**323**
LungSquaCarc	16	6,829	3,868	53	57	**25**
LungSmallCarc	28	8,883	5,746	58	68	**17**
Lymphoma B-Cell	37	6,383	**1,754**	2,238	2,284	2,252
Medulloblastoma	14	6,692	1,123	265	247	**230**
Melanoma	31	6,719	2,467	459	365	**233**
Multiple Myeloma	7	5,165	**2,871**	3,754	3,871	3,683
Ovarian	22	6,481	2,632	724	627	**593**
PancAdeno	13	3,140	1,777	140	**118**	123
ProstAdeno	7	6,565	2,345	457	**371**	514
Rhabd	6	4,871	1,957	181	111	**26**
Soft-Tissue Sarcoma	23	8,572	4,447	2,008	1,992	**1,970**
StomAdeno	17	6,530	2,878	331	**319**	322
ThyrCarc	8	10,352	2,834	**1,222**	1,325	1,538

For 27 cancer types (rows) with a given number of known TSG (second column), this table shows the performance in terms of cross-validate *CE* of different methods for TSG prediction, including DiffMut (column 3) and different variants of single- and multitask LOTUS (columns 4 to 7). ALL stands for Acute Lymphocytic Leukemia, BladUroCarc for Bladder Urothelial Carcinoma, BreastAdeno for Breast Adenocarcinoma, CLL for Chronic Lymphocytic Leukemia, EndomCarc for Endometrial Carcinoma, GliobMulti for Glioblastoma Multiform, HNSC for Head and Neck Squamous Cell Carcinoma, LAML for Acute Myeloid Leukemia, LiverHepCarc for Liver Hepatocellular Carcinoma, LungAdeno for Lung Adenocarcinoma, LungSquaCarc for Lung Squamous Cell Carcinoma, LungSmallCarc for Lung Small Cell Carcinoma, PancAdeno for Pancreatic Adenocarcinoma, ProstAdeno for Prostate Adenocarcinoma, Rhabd for Rhabdomyosarcoma, StomAdeno for Stomach Adenocarcinoma and ThyrCarc for Thyroid Carcinoma.

**Table 13:** Cross-validated *CE* for DiffMut and LOTUS prediction of disease specific OGs in the single- and multitask settings.

Disease	Number of OGs	DiffMut	Single-Task LOTUS	Aggregation LOTUS	Multitask LOTUS	Multitask LOTUS2
ALL	52	8,479	2,649	1,232	1,269	**1,145**
Astrocytoma	13	7,847	2,894	75	63	**13**
BladUroCarc	10	5,324	1,578	210	**139**	140
BreastAdeno	19	2,672	1,371	852	806	**792**
CLL	19	4,582	3,821	1,537	1,501	**1,462**
Colorectal	23	4,043	3,376	818	784	**758**
EndomCarc	8	5,112	1,671	122	128	**105**
GliobMulti	22	4,915	2,539	143	128	**106**
HNSC	23	4,539	2,917	**1,305**	1,500	1,504
Kidney Cancer	11	5,774	1,903	**543**	600	763
LAML	56	4,990	2,623	1,418	1,408	**1,307**
Low-Grade Glioma	10	3,753	1,541	46	33	**3**
LungAdeno	26	4,510	2,038	84	79	**40**
LungSmallCarc	6	3,243	2,129	1,061	**666**	864
LungSquaCarc	24	5,737	1,641	67	54	**17**
Lymphoma B-Cell	34	4,765	2,424	1,712	1,714	**1,669**
Medulloblastoma	5	7,165	58	93	34	**25**
Melanoma	35	3,377	1,925	1,576	1,550	**1,525**
Multiple Myeloma	9	3,466	2,870	**1,823**	1,877	2,026
Neuroblastoma	5	5,298	3,830	2,078	**2,077**	2,101
Ovarian	12	6,371	3,606	1,256	**869**	870
PancAdeno	6	1,464	1,142	498	426	**302**
ProstAdeno	13	6,523	2,451	**955**	1,599	1,475
Rhabd	7	8,265	1,978	172	104	**30**
Soft-Tissue Sarcoma	38	8,886	2,480	**2,424**	2,466	2,444
StomAdeno	10	2,235	750	**85**	127	97
ThyrCarc	8	8,407	2,656	547	612	**494**

For 27 cancer types (rows) with a given number of known OG (second column), this table shows the performance in terms of cross-validate *CE* of different methods for OG prediction, including DiffMut (column 3) and different variants of single- and multitask LOTUS (columns 4 to 7). The abbreviations of cancer types are explained in the legend of Table 12.

For most diseases (25/27 for TSG, 27/27 for OG), single-task LOTUS and DiffMut lead to the worst *CE*, confirming the difficulty to treat each cancer type individually, due to the small number of known driver genes and to the smaller mutation database available for each cancer type type. Interestingly, Aggregation LOTUS often leads to a strong improvement in performance. This shows that different cancer types often share some common mechanisms and driver genes, and therefore, simply using all the available information as in a pan-cancer paradigm improves the performance of driver gene prediction for each disease. However, in many cases, the multitask LOTUS and LOTUS2 algorithms lead to an additional improvement over Aggregation LOTUS, LOTUS2 leading in general to the best results (in 17 types out of 27 for TSG prediction, and in 17 types out of 27 for OG prediction). On average, the decrease in *CE* between Aggregate LOTUS and LOTUS2 is of 20% for OG and 18% for TSG. The improvement in performance observed between Aggregate LOTUS and LOTUS2 shows that, besides some driver mechanisms common to many cancers, each cancer presents some specific driver mechanisms that can only be captured by prediction methods able to integrate some biological knowledge about the different diseases. The above results show that multitask algorithms allowing to share information between cancers according to their biological similarities such as LOTUS2, rather than on more naive rules, better capture these specific driver genes. They also show that the kernel *K*_*diseases*_ = *K*_*descriptors*_ built on disease descriptors contains some relevant biological information to compare diseases.

To measure how different the predictions of LOTUS2 are for each cancer type, we compared the first 50 predictions for each type. Aggregating all predictions for TSGs (respectively OGs) results in 210 genes (respectively 224 genes), which shows that various cancer types share some drivers, but that the prediction lists are different. Indeed, some drivers with high penetrance (such as TP53) are expected to be found in most cancer types, whereas other drivers are more specific to given organs or cell types, in particular since all genes are not expressed in all cell types.

In addition, multitask algorithms based on task descriptors (here, disease descriptors) appear to be promising in order to include prior knowledge about diseases and share information according to biological features characterizing the diseases.

Finally, note that we did not try to run TUSON, MutSigCV, MUFFINN or 20/20+ to search for cancer specific driver genes in the single-task setting (they cannot be run in the multi-task setting). Indeed, according to the results of pan-cancer studies in the single-task setting, they do not perform as well as single-task LOTUS. Considering that single-task LOTUS and DiffMut were far from reaching the performance of multi-task LOTUS for prediction of cancer specific driver genes, TUSON, MutSigCV, MUFFINN and 20/20+ are not expected to reach these performance either.

## Discussion

Our work demonstrates that LOTUS outperforms several state-of-the-art methods on all tested situations for driver gene prediction. This improvement results from various aspects of the LOTUS algorithm. First, LOTUS allows to include the PPI network information as independent prior biological knowledge. In the single-task setting, we proved that this information has significance for the prediction of cancer driver genes. Because LOTUS is based on kernel methods, it is well suited to integrate other data from multiple sources such as protein expression data, information from chip-seq, HiC or methylation data, or new features for mutation timing as designed in [69]. Further development could involve the definition of other gene kernels based on such type of data, and combine them with our current gene kernel, in order to evaluate their relevance in driver gene prediction.

We also showed how LOTUS can serve as a multitask method. It relies on a disease kernel that controls how driver gene information is shared between diseases. Interestingly, we showed that building a kernel based on independent biological prior knowledge about disease similarity leads on average to the best prediction performance with respect to single-task algorithms, and also with respect to a more generic and naive multitask learning strategy that does not incorporate knowledge about the cancer types. Again, the kernel approach leaves space for integration of other types and possibly more complex biological sources of information about diseases. Our multitask approach thus allows to make prediction for cancer types with very few known driver genes, which would be less reliable with the single-task methods. We considered here only diseases with at least 4 known driver genes, in order to perform cross-validation studies, which was necessary to evaluate the methods. However, it is important to note that in real-case studies, at the extreme, both versions of multitask LOTUS could make driver gene prediction for the 30 cancer types, including those for which no driver gene is known.

LOTUS is a machine learning algorithm based on one-class SVM. In fact, the most classical problem in machine learning is binary classification, where the task is to classify observations into two classes (positives and negatives), based on training sets P of known positives and N of known negatives. Driver gene detection can be seen as binary classification of TSGs vs. neutral genes, and of OGs vs. neutral genes. However, although the P set is composed of known driver genes, it is not straightforward to build the N set because we cannot claim that some genes cannot be drivers. Thus, driver gene detection should rather be seen as binary classification problem with only one training set P of known positives. This problem is classically called PU learning (for Positive-Unknown), as opposed to PN learning (for Positive-Negative).

The classical way to solve PU learning problems is to choose a set N of negatives among the unlabeled data and apply a PN learning method. For example, one can consider all unknown items as negatives (some of which may be reclassified afterwards as positives), or randomly choose bootstrapped sets of negatives among the unknown, like in [34]. Both methods assume that a minority of the unlabeled items are in fact positives, which is expected for driver genes.

The one-class SVM algorithm [70] can also be used as a PU learning method, in which a virtual item is chosen as the training set of negatives. We preferred this approach because in preliminary studies, we found that it had slightly better performances than PU learning methods and was also faster.

For LOTUS, as for all machine learning algorithm, the set of known driver genes is critical: if this set is poorly chosen (*i.e.*, if some genes were wrongly reported as driver genes, or more likely, if the reported genes are not the best driver genes), the best algorithm might not minimize the *CE*. To circumvent this problem, we propose two new approaches for future developments: one could build a multi-step algorithm that iteratively removes some genes from the positive set and labels them as unknown, and relabel as positives some of the best ranked unknown genes. We believe that such an algorithm would make the set of positives converge to a more relevant list. Alternatively, one could assign (finite) scores to the known driver genes before performing classification and increment these scores at each step.

## Materials and methods

### Pan-cancer LOTUS

LOTUS is a new machine learning-based method to predict new cancer driver genes, given a list of know ones. In the simplest, pan-cancer setting, we consider a list of *N* known driver genes {*g*_1_, …, *g*_*N*_}, and the goal of LOTUS is to learn from them a scoring function *f*(*g*), for any other gene *g*, that predicts how likely it is that *g* is also a cancer gene. Since TSGs and OGs have different characteristics, we treat them separately and build two scoring functions *f*_*TSG*_ and *f*_*OG*_ that are trained from lists of know TSGs and OGs, respectively.

LOTUS learns the scoring function *f*(*g*) with a one-class support vector machine (OC-SVM) algorithm [70], a classical method for novelty detection and density level set estimation [71]. The scoring function *f*(*g*) learned by a OC-SVM given a training set {*g*_1_, …, *g*_*N*_} of known cancer genes takes the form:

$$\begin{array}{c} f\left(g\right)=\sum_{i=1}^{N}\alpha_{i}K\left(g_{i},g\right)\;, \end{array}$$ (1)

where *α*_1_, …, *α*_*N*_ are weights optimized during the training of OC-SVM [70], and *K*(*g*, *g*′) is a so-called *kernel* function that quantifies the similarity between any two genes *g* and *g*′. In other words, the score of a new gene *g* is a weighted combination of its similarities with the known driver genes.

The kernel *K* encodes the similarity among genes. Mathematically, the only constraint that *K* must fulfill is that it should be a symmetric positive definite function [30]. This leaves a lot of freedom to create specific kernels encoding prior knowledge about relevant information to predict driver genes. In addition, one can easily combine heterogeneous information in a single kernel by, e.g., summing two kernels based on different sources of data. In this work, we restrict ourselves to the following basic kernels, and leave for future work a more exhaustive search of optimization of kernels for cancer gene prediction.

- *Mutation kernel*. Given a large data set of somatic mutations in cohorts of cancer patients, we characterize each gene *g* by a vector $\Phi_{mutation}\left(g\right)\in R^{3}$ encoding 3 features. For OG prediction the three features are the number of damaging missense mutations (defined as in [26] as mutations with a Polyphen2 score larger than 0.447), the total number of missense mutations, and the entropy of the spatial distribution of the missense mutations on each gene. For TSG prediction, the features are the number of frameshift mutations, the number of LOF mutations (defined as the nonsense and frameshift mutations), and the number of splice site mutations. These features were calculated as proposed by [26]. We chose them because they were found to best discriminate OGs and TSGs by the TUSON algorithm [26] and were also all found among the most important features selected by the random forest algorithm used by the 20/20+ method [28]. Given two genes *g* and *g*′ represented by their 3-dimensional vectors Φ(*g*) and Φ(*g*′), we then define the mutation kernel as the inner product between these vectors:
  $$\begin{array}{c} K_{mutation}\left(g,g^{\prime }\right)=\Phi_{mutation}{\left(g\right)}^{⊤}\Phi_{mutation}\left(g^{\prime }\right)\;. \end{array}$$
  Notice that using *K*_*mutation*_ as a kernel in OC-SVM produces a scoring function (1) which is simply a linear combination of the three features used to define the vector Φ_*mutation*_.
- *PPI kernel*. Given an undirected graph with genes as vertices, such as a PPI network, we define a PPI kernel *K*_*PPI*_ as a graph kernel over the network [72, 73]. More precisely, we used a diffusion kernel of the form *K*_*PPI*_ = exp_*M*_(−*L*), where *L* = *I* − *D*^−1/2^ *AD*^−1/2^ is the normalized Laplacian of the graph and exp_*M*_ is the matrix exponential function. Here *I* is the identity matrix, *A* stands for the adjacency matrix of the graph (*A*_*i*,*j*_ = 1 if vertices *i* and *j* are connected, 0 otherwise) and *D* for the diagonal matrix of degrees ($D_{ii}=\sum_{j=1}^{n}A_{ij}$). Intuitively, two genes are similar according to *K*_*PPI*_ when they are close and well connected through several routes to each other on the PPI network, hence learning a OC-SVM with *K*_*PPI*_ allows to diffuse the information about cancer genes over the network.
- *Integrated kernel*. In order to train a model that incorporates informations about both mutational features and PPI, we create an integrated gene kernel by simply averaging the mutation and PPI kernels:
  $$\begin{array}{c} K_{gene}\left(g,g^{\prime }\right)=(K_{mutation}\left(g,g^{\prime }\right)+K_{PPI}\left(g,g^{\prime }\right))/2\;. \end{array}$$
  While more complex kernel combination strategies such as multiple kernel learning could be considered, we restrict ourselves to this simple kernel addition scheme to illustrate the potential of our approach for heterogeneous data integration.

### Multitask LOTUS for cancer type-specific predictions

The pan-cancer LOTUS approach can also be used for cancer-specific predictions, by restricting the training set of known cancer driver genes to those genes known to be driver in a particular cancer type. However, for many cancer types, only few driver genes have been validated, creating a challenging situation for machine learning-based methods like LOTUS that rely on a training set of known driver genes to learn a scoring function. Since cancer driver genes of different cancer types are likely to have similar features, we propose instead to learn jointly cancer type-specific scoring functions by sharing information about known driver genes across cancer types, using the framework of multitask learning [33, 34]. Instead of starting from a list of known driver genes, we now start from a list of known (cancer gene, cancer type) pairs of the form {(*g*_1_, *d*_1_), …, (*g*_*N*_, *d*_*N*_)}, where a sample (*g*_*i*_, *d*_*i*_) means that gene *g*_*i*_ is a known cancer gene in disease *d*_*i*_. Note that a given gene (and a given cancer type) may of course appear in several such pairs.

The extension of OC-SVM to the multitask setting is straightforwardly obtained by creating a kernel for (gene, disease) pairs of the form:

$$\begin{array}{c} K_{pair}(\left(g,d\right),\left(g^{\prime },d^{\prime }\right))=K_{gene}\left(g,g^{\prime }\right)\times K_{disease}\left(d,d^{\prime }\right)\;, \end{array}$$

where *K*_*gene*_ is a kernel between genes such as that used in pan-cancer LOTUS and *K*_*disease*_ is a kernel between cancer types described below. We then simply run the OC-SVM algorithm using *K*_*pair*_ as kernel and {(*g*_1_, *d*_1_), …, (*g*_*N*_, *d*_*N*_)} as training set, in order to learn a cancer type-specific scoring function of the form *f*(*g*, *d*) that estimates the probability that *g* is a cancer gene for cancer type *d*.

The choice of the disease kernel *K*_*disease*_ influences how information is shared across cancer types. One extreme situation is to take the uniform kernel *K*_*uniform*_(*d*, *d*′) = 1 for any *d*, *d*′. In that case, no distinction is made between diseases, and all known cancer driver genes are pooled together, recovering the pan-cancer setting (with the slight difference that genes may be counted several times in the training set if they appear in several diseases). Another extreme situation is to take the Dirac kernel *K*_*Dirac*_(*d*, *d*′) = 1 if *d* = *d*′, 0 otherwise. In that case, no information is shared across cancer types, and the joint model over (gene, disease) pairs is equivalent to learning independently a model for each disease, as in the single-task approach.

In order to leverage the benefits of multitask learning and learn disease-specific models by sharing information across diseases, we consider instead the following two disease kernels:

- First, we consider the standard multitask learning kernel:
  $$\begin{array}{c} K_{multitask}\left(d,d^{\prime }\right)=(K_{uniform}\left(d,d^{\prime }\right)+K_{Dirac}\left(d,d^{\prime }\right))/2\;, \end{array}$$
  which makes a compromise between the two extreme uniform and Dirac kernels [33]. Intuitively, for a given cancer type, prediction of driver genes is made by assigning twice more weight to the data available for this cancer than to the data available for all other cancer types.
- Second, we test a more elaborate multitask version where we implement the idea that a given cancer might share various degrees of biological similarities with other cancers. Therefore, known driver genes for other cancers should be shared with those of the considered cancer based on this similarity. Hence, we create a specific disease kernel *K*_*cancer*_(*d*, *d*′) to capture how similar two cancer types are. To create *K*_*cancer*_, we first represent each cancer type as a 43-dimensional binary vector as follows. The first 12 bits correspond to a list of cancer type characteristics used in COSMIC to describe tumors: adenocarcinoma, adenoma, blastoma, carcinoma, glioma, leukemia, lymphoma, medulloblastoma, melanoma, myeloma, rhabdomyosarcoma, sarcoma. The last 31 components correspond to localization characteristics also used in COSMIC to describe tumors: adrenal glands, astrocytes, B-cell, bladder, bone, breast, cervix, central nervous system, colon, ducts, endometrium, eye, head and neck, heart, kidney, liver, lung, lymphocytes, mucosa, muscle, nerve, oesophagus, ovary, pancreas, prostate, salivary glands, skin, soft tissue, squamous cell, stomach, T-cell, thyroid. A disease might be assigned one or several types and be associated to one or several locations. For example, Melanoma is associated with a single type (“melanoma”) and four localizations (“skin”, “mucosa”, “eye” and “head and neck”), so that Melanoma is described by a vector with five 1’s and thirty-eight 0’s. For each disease, we construct the list of binary features by documenting every disease in the literature. The corresponding vectors encoding the considered disease are given in S3 Table. Finally, if $\Psi \left(d\right)\in R^{43}$ denotes the binary vector representation of disease *d*, we create the disease kernel as a simple inner product between these vectors, combined with the standard multitask kernel, i.e.:
  $$\begin{array}{c} K_{cancer}\left(d,d^{\prime }\right)=(\Psi {\left(d\right)}^{⊤}\Psi \left(d^{\prime }\right)+K_{uniform}\left(d,d^{\prime }\right)+K_{Dirac}\left(d,d^{\prime }\right))/3\;. \end{array}$$

### Data

When comparing LOTUS to TUSON and MUFFINN, we use a dataset of somatic mutations collected from COSMIC [14], TCGA (http://cancergenome.nih.gov/) and [18], that was used in [26]. This dataset contains a total of 1, 195, 223 mutations across 8, 207 patients affecting 18, 843 genes.

When comparing LOTUS to DiffMut and 20/20+, we use a dataset of somatic mutations borrowed from [28]. This dataset contains a total of 729, 205 mutations across 7, 916 patients affecting 19, 320 genes.

When comparing LOTUS to MutSigCV, we use an example dataset available on GenePattern. This dataset contains a total of 137, 343 mutations across 177 patients of lung squamous cell carcinoma affecting 16, 885 genes.

We obtained the PPI network from the HPRD database release 9 from April 13, 2010 [74]. It contains 39, 239 interactions among 7, 931 proteins. As for known pan-cancer driver genes, we consider three lists in our experiments: (i) the TUSON train set, proposed in [26], consists of two high confidence lists of 50 OGs and 50 TSGs extracted from CGC (release v71) based on several criteria, in particular excluding driver genes reported through translocations; (ii) the 20/20 train set, proposed in [28] to train the 20/20+ method, contains 53 OGs and 60 TSGs; finally, (iii) the CGCv86 train set consists of two broader lists that we extracted from CGC release v86 of the COSMIC database: we consider as OGs the genes annotated as “oncogene”, “oncogene, TSG”, “oncogene, fusion”, “oncogene, TSG, fusion”, and as TSGs the genes annotated as “TSG”, “oncogene, TSG”, “TSG, fusion”, “oncogene, TSG, fusion”. For cancer type-specific lists of driver genes, we only consider the CGCv86 train sets. We distinguished 30 diseases based on the available annotations describing patients in the mutation matrix, only merging “Kidney Chromophobe”, “Kidney Papillary Cell Carcinoma” and “Kidney Clear Cell Carcinoma” into “Kidney Cancer”, “DLBCL” and “Lymphoma B-Cell” into “Lymphoma B-Cell” and neglecting the unspecific “CARC”. The names of these diseases and their numbers of associated TSGs and OGs can be found in S2 Table. For each of the resulting diseases, 0 to 56 TSGs/OGs were known in CGCv86. We considered only diseases with at least 4 known TSGs or OGs available, in order to have enough learning data points to perform a two-fold cross-validation scheme, which led us to consider 27 diseases for TSG prediction and 27 for OG prediction.

### Comparing different methods

Among LOTUS, TUSON, 20/20+, DiffMut, MUFFINN and MutSigCV, we can distinguish on the one side, the unsupervised methods MutSigCV, MUFFINN and DiffMut that score candidate genes independently of any training set of known drivers, and the supervised methods LOTUS, TUSON and 20/20+ that make predictions based on a training set of known driver genes.

In addition, all methods use gene descriptors that are calculated based on a mutation databases, and therefore, changing the mutation database will change the prediction performance.

In order to make fair comparison between LOTUS and the other five methods, we performed several experiments in which LOTUS is trained with the training set of the TUSON (respectively 20/20+) paper when compared to TUSON and MUFFINN (respectively 20/20+). In addition, in all experiments, the gene features calculated for LOTUS and MUFFINN were based on the same mutation databases as those used by the other methods in their respective papers.

Therefore, for a fair comparison between LOTUS, MUFFINN and TUSON, we use the mutation database available on the website of the authors along with their training sets of OGs and TSGs provided in [26]. We evaluate the performance of LOTUS on this dataset by 5-fold cross-validation repeated twice (see Methods). For TUSON, we use the prediction results available in [26] and evaluate the *CE* as the mean number of non-driver genes that are ranked before known driver genes of the TUSON train sets. Finally, we downloaded and re-ran MUFFINN.

For a fair comparison between LOTUS and 20/20+, we use the mutation database of 20/20+ and the training sets of OGs and TSGs provided by the authors on their website [28]. We evaluate the performance of LOTUS as above. However we note that the 20/20+ score itself is obtained by a bootstrap procedure similar to our cross-validation approach [28].

For a fair comparison between LOTUS and MutSigCV, we use the example mutation database available only for lung squamous cell tumours. Since MutSigCV does not use a train set of driver genes, we trained LOTUS with known OGs and TSGs available in CGCv86 for lung squamous cell tumours. MutSigCV provides a ranked list of genes that does not distinguish TSG and OG. Therefore, the *CE* is obtained by averaging the numbers of non-driver genes ranked before each driver genes in the train sets used for LOTUS.

Finally, for a fair comparison between LOTUS and DiffMut, we use the 20/20+ mutation dataset for both methods. LOTUS is trained with the 20/20+ training sets of OGs and TSGs. We run DiffMut using the latest version of the algorithm, and we compute the *CE* related to the 20/20+ training sets of OGs and TSGs.

### Experimental protocol

To assess the performance of a driver gene prediction method on a given gold standard of known driver genes, we score all genes in the COSMIC database and measure how well the known driver genes are ranked. For that purpose, we plot the ROC curve, considering all known drivers as positive examples and all other genes in COSMIC as negative ones, and define the consistency error (*CE*) as

$$\begin{array}{c} CE=\#N\times (1-AUC), \end{array}$$

where #N is the number of negative genes, and *AUC* denotes the area under the ROC curve. In other words, *CE* measures the mean number of “non-driver” genes that the prediction method ranks higher than known driver genes. Hence, a perfect prediction method should have *CE* = 0, while a random predictor should have a *CE* near #N/2.

To estimate the performance of a machine learning-based prediction method that estimates a scoring function from a training set of known driver genes, we use *k*-fold cross-validation for each given gold standard set of known driver genes. In *k*-fold cross-validation, the gold standard set is randomly split into *k* subsets of roughly equal sizes. Each subset is removed from the gold standard in turn, the prediction method is trained on the remaining *k* − 1 subsets, and its *CE* is estimated considering the subset left apart as positive examples, and all other genes of COSMIC not in the gold standard set as negative examples. A mean ROC curve and mean *CE* is then computed from the *k* resulting ROC curves. This computation is repeated several times to consider several possibly different partitions of the gold standard set.

### Tuning of parameters

Each version of LOTUS depends on a unique parameter, the regularization parameter *C* of the OC-SVM algorithm. Each time a LOTUS model is trained, its *C* parameter is optimized by 5-fold cross-validation on the training set, by picking the value in a grid of candidate values {2^−5/2^, 2^−4/2^, …, 2^5/2^} that minimizes the mean *CE* over the folds.

### Other driver prediction methods

We compare the performance of LOTUS to five other state-of-the-art methods: MutSigCV [21], which is a frequency-based method, TUSON [26] and 20/20+ [28] that combine frequency and functional information, MUFFINN [32] and DiffMut that analyses mutation profiles on genes.

MutSigCV searches driver genes among significantly mutated genes which adjusts for known covariates of mutation rates. The method estimates a background mutation rate for each gene and patient, based on the observed silent mutations in the gene and noncoding mutations in the surrounding regions. Incorporating mutational heterogeneity, MutSigCV eliminates implausible driver genes that are often predicted by simpler frequency-based models. For each gene, the mutational signal from the observed non-silent counts are compared to the mutational background. The output of the method is an ordered list of all considered genes as a function of a p-value that estimates how likely this gene is to be a driver gene.

TUSON uses gene features that encode frequency mutations and functional impact mutations. The underlying idea is that the proportion of mutation types observed in a given gene can be used to predict the likelihood of this gene to be a cancer driver. After having identified the most predicting parameters for OGs and TSGs based on a train set (called the TUSON train set in the present paper), TUSON uses a statistical model in which a p-value is derived for each gene that characterizes its potential as being an OG or a TSG, then scores all genes in the COSMIC database, to obtain two ranked lists of genes in increasing orders of p-values for OGs and TSGs.

The 20/20+ method encodes genes based on frequency and mutation types, and other biological information. It uses a train set of OGs and TSGs (called the 20/20 train set in the present paper) to train a random forest algorithm. Then, the random forest is used on the COSMIC database and the output of the method is again a list of genes ranked according to their predicted score to be a driver gene [28]. We did not implement this method, so we decided to evaluate its performance only on its original training set: the 20/20 dataset. Moreover, we applied the same method to compute the *CE* as for MutSigCV and TUSON, which should actually give an advantage to 20/20+, since it is harder to make predictions in a cross-validation loop using a smaller set of known driver genes.

DiffMut uses a dataset of somatic mutations and a dataset of healthy genomes, but no training sets of known driver genes. It compares the mutation profiles on a gene in the mutation dataset with the nucleotide variation profile in the healthy genomes, and computes for every gene a score that allows to rank all genes according to their potential as OG or TSG.

MUFFINN uses a dataset of somatic mutations and extracts the number of non-synonymous mutations per gene. Then, it computes a score (either DNmax or DNsum) and propagates these scores on a functional gene network (either HumanNet or STRING). The final scores are used to compute four different rankings for all genes. Among these four possibilities, we systematically used the version that yields the best result for MUFFINN,. Note however that, in practice, the user would not know which version should be preferred.

### Code and data availability

We implemented LOTUS and performed all experiments in R using in particular the kernlab package for OC-SVM [75]. The code and data to reproduce all experiments are available at https://github.com/LOTUSproject/LOTUS.

## Supporting information

S1 Table TSG and OG rankings for LOTUS with the 20/20, the TUSON and the CGC v86 datasets. Note that the training sets were removed every time.
(XLSX)

S2 Table List of cancer types. Cancer types derived from annotations in the 20/20 mutation dataset along with their numbers of associated OG and TSG.
(XLSX)

S3 Table Description of cancer types. Descriptors of all cancer types according to their localizations and types that are used to compute the disease kernel used by LOTUS2.
(XLSX)
